# Mesenchymal Stem Cells Derived from Dental Pulp: A Review

**DOI:** 10.1155/2016/4709572

**Published:** 2015-12-08

**Authors:** Edgar Ledesma-Martínez, Víctor Manuel Mendoza-Núñez, Edelmiro Santiago-Osorio

**Affiliations:** ^1^Hematopoiesis and Leukemia Laboratory, Research Unit on Cell Differentiation and Cancer, FES Zaragoza, National Autonomous University of Mexico, 09230 Mexico City, MEX, Mexico; ^2^Research Unit on Gerontology, FES Zaragoza, National Autonomous University of Mexico, 09230 Mexico City, MEX, Mexico

## Abstract

The mesenchymal stem cells of dental pulp (DPSCs) were isolated and characterized for the first time more than a decade ago as highly clonogenic cells that were able to generate densely calcified colonies. Now, DPSCs are considered to have potential as stem cell source for orthopedic and oral maxillofacial reconstruction, and it has been suggested that they may have applications beyond the scope of the stomatognathic system. To date, most studies have shown that, regardless of their origin in third molars, incisors, or exfoliated deciduous teeth, DPSCs can generate mineralized tissue, an extracellular matrix and structures type dentin, periodontal ligament, and dental pulp, as well as other structures. Different groups worldwide have designed and evaluated new efficient protocols for the isolation, expansion, and maintenance of clinically safe human DPSCs in sufficient numbers for various therapeutics protocols and have discussed the most appropriate route of administration, the possible contraindications to their clinical use, and the parameters to be considered for monitoring their clinical efficacy and proper biological source. At present, DPSC-based therapy is promising but because most of the available evidence was obtained using nonhuman xenotransplants, it is not a mature technology.

## 1. Introduction

The regenerative capacity of adult tissues depends on their stem cell populations, which stably self-renew and, in turn, give rise to progeny that possess the ability to differentiate into specialized cells. Stem cells have different names depending on the tissue of origin; thus there are hematopoietic, mesenchymal, endothelial, mammary, intestinal, neural, skin, muscle, and hair follicle stem cells, among others.

Among these stem cells, mesenchymal stem cells (MSCs) are noteworthy for their pluripotency, which means that they can differentiate into cells of any type, including those of the three embryonic germ layers. Because of their capacity for differentiation and wide tissue distribution and because their infusion has induced tissue repair in both preclinical and clinical models, MSCs are very attractive tools for tissue repair. Therefore, MSCs of dental origin have been tested as candidates for cellular therapy of stomatognathic disorders, such as periodontal disease (PD), and for maxillofacial reconstruction. In particular, it has been shown that human dental pulp stem cells (DPSCs) can generate mineralized tissue, an extracellular matrix and structures type dentin, dental pulp, and periodontal ligament in xenograft models. Herein, we review the general characteristics and immunophenotypes that define the DPSCs as MSCs, their isolation and cultivation, and their potential applications to tissue repair, emphasizing the possible administration routes, type of scaffold to use, and suggestions for their clinical applications.

## 2. Dental Pulp Stem Cells: General Characteristics

Teeth develop due to interactions between the oral ectodermal epithelial cells and MSCs, first forming the enamel organ and the second forming papilla and the dental follicle. MSCs give rise to other components of the tooth, such as dentin, pulp, cementum, and the periodontal ligament [1].

The presence of different types of MSC populations in teeth has been described, which depending on the harvest site are called dental pulp stem cells (DPSCs), periodontal ligament stem cells (PDLSCs), apical papilla stem cells (SCAPs), dental follicle stem cells (DFSCs), and gingival tissue stem cells (GMSCs) [2], although they are generically referred to as dental stem cell (DSCs). This set of stem cells is particularly interesting because teeth, despite their small size, are a source of abundant cells for therapeutic procedures, and their preparation can be linked to routine tooth extraction, which does not place an additional burden on the patient [3].

However, some authors suggested that this broad heterogeneity of DSCs could be a drawback for clinical applications if the cellular origin is not identifiable because different subpopulations of DSCs may have different potentials for proliferation and differentiation that could prevent obtaining perfectly predictable and reproducible results [4].

DPSCs, also known as postnatal dental pulp stem cells, were first isolated by Gronthos et al. from third molars and were characterized as cells with a high level of clonogenicity and proliferation and the ability to generate densely calcified colonies and occasional nodules [5]. The identity of the DPSCs as MSCs has been confirmed by their ability to differentiate into neural ectodermal cells and adipocytes, odontoblasts, osteoblasts, chondrocytes, and myoblast cells of mesodermal origin, confirming their plasticity [6].

These cells are located within the dental crown, in a “niche sealing” or “pulp chamber” that houses the connective tissue known as pulp. The resident tissue cells are a heterogeneous population represented by stromal fibroblasts also known as pulpoblasts [7] and accompanied by odontoosteoprogenitor populations, neural, vascular cells and inflammatory immune cells such as granulocyte and macrophage cells [8].

During embryonic development, the dental pulp is a tissue that some authors have described as “ectomesenchyme” because it is derived from ectodermal cells that grow at the periphery of the neural tube, migrate to the oral region, and then differentiate into cells of the mesenchymal phenotype [9]. The epithelial cells give rise to enamel forming ameloblasts, and the MSCs form odontoblasts, pulp, and periodontal ligament [10].

Functionally, the dental pulp is responsible for the maintenance and repair of the periodontal tissue and its associated immune system, has a high regenerative capacity, and responds to various types of damage. For example, in cases of severe irritation caused by deep caries or restorative procedures leading to the destruction of the layer of odontoblasts or pulp progenitors (DPSCs), dental pulp cells proliferate and migrate into the damaged tissue to differentiate into odontoblast and form reparative dentin [11, 12] which has been proposed to be the main mechanism leading to reparative dentinogenesis [13].

## 3. Immunophenotype

The Committee of Mesenchymal Stem Cells and Tissues of the International Society for Cellular Therapy (ISCT) proposed in 2005 that 95% of human MSCs express at least the surface antigens CD105 (endoglin), CD73 (5′-ectonucleotidase), and CD90/Thy-1 (glycosylphosphatidylinositol-anchored glycoprotein) but do not express the (≤2%+) CD11b, CD14, CD19, CD34, CD45, CD79a, and HLA-DR surface antigens. However, several groups have proposed other cell surface antigens to prospectively isolate MSCs, for example, STRO-1 (stromal precursor antigen 1), VCAM-1 (vascular cell adhesion molecule 1), SH2 (Src homology 2), SH3/SH4, CD271, GD2 (ganglioside 2), and SSEA4 (stage-specific embryonic antigen-4) [14–18]. Thus, there are discrepancies and inconsistencies regarding other antigens that are expressed by MSCs in addition to CD73, CD90, and CD105 (Table 1). As is currently the case for MSCs, DPSC do not seem to express a marker that exclusively identifies them [19, 20]; in fact, some groups have proposed that DPSCs might have an immunophenotype difference from that previously described by the ISCT to MSCs.

One possible reason for the apparent differential expression of antigens on DPSCs is the presence of different subpopulations of MSCs in dental pulp that have different biological activities. For example, highly proliferative DPSCs tend to express CD44+, CD90+, and CD166+ [23]. MSC dental pulp STRO-1+ are a subset of DPSCs with odontoosteogenic properties, whereas DPSC CD34+, CD117+, and CD45− have a greater capacity for self-renewal and osteogenic differentiation and generate autologous fibrous-bone tissue in vitro and bone tissue when they are implanted in mice [33]. Other markers of DPSCs that seem to be related to the stemness of MSCs are CD29+, CD44+, and CD73+ [28, 34, 38] which, however, are not relevant to isolate DPSC [39].

Furthermore, some authors have suggested that the expression of genes as OCT-4 and NANOG, both of which are transcription factors involved in the maintenance of the multi/pluripotency [37], can be used to identify MSCSs [40]. However, despite the increase in the number of new DPSC markers, the reality is that most basic and preclinical studies are using the immunophenotype proposed by the ISCT for identifying MSCs.

Interestingly, it has been shown that, like other stem cells, DPSCs have the ability to exclude the fluorescent DNA-binding dye Hoechst33342, in what is known as a “side population” (SP). Exploiting this phenomenon, a population of adult stem cells with the ability to differentiate into osteoblasts, chondroblasts, adipocytes, and neuronal cells was isolated from dental pulp [41], and these cells were found to be negative for hematopoietic antigens, such as CD146 and CD31, and possessed the ability to induce angiogenesis and vasculogenesis during tissue repair [42]. However, some studies have shown that the SP cells lacked the properties of stem cells. Moreover, their clinical application must be approached with caution because the Hoechst dye binds to DNA and is a possible carcinogen, some studies have shown that this dye can affect cellular differentiation, and it is a potential tumorigenic-clonogenicity enhancer [23].

## 4. Heterogeneity of DPSCs

DPSCs are stem cells derived from human exfoliated deciduous teeth (SHED), from permanent secondary dentition systems (properly known as DPSCs), from teeth extracted by orthodontist due to impaction or irreversible periodontitis, or from inflamed pulp tissue [2, 43].

SHED cells were isolated by Miura et al. from primary dentition systems and were characterized as cells with a high proliferation rate and the ability to differentiate into osteoblasts, neural cells, adipocytes, and odontoblasts. As in the case of DPSCs obtained from permanent teeth, SHED cells can generate tissue pulp/dentin and bone cell type [44], with different levels of odontoblast-like cells and dentin mineralized when transplanted into immunodeficient mice in a hydroxyapatite tricalcium phosphate (HA/TCP) scaffold [45].

It has been suggested that SHED cells have a proliferative ability greater than that of DPSCs isolated from third molars, incisors, or supernumerary teeth or that of bone marrow-derived MSCs, because they represent a more immature stem cell population [46, 47]. Thus, as bone marrow-derived MSCs and umbilical cord-derived MSCs have been show to differ, in the case of dental pulp-derived MSCs, it has been shown that the age of the teeth that served as the source of the dental pulp tissues imparted different characteristics and propensities toward differentiating along a specific lineage [48]. The studies of Suchánek et al. showed that SHED cells have a higher doubling time than DPSCs which is consistent with their cell cycle distribution, wherein 69.8% of the SHED cells were in S and G2 stage, but only 56% of the DPSCs were in that phase [22]. Furthermore, the surface antigen profile of SHED cells has been shown to differ from those of DPSC and bone marrow-derived MSC. Because proliferation-related and extracellular matrix formation genes, such as those encoding transforming growth factor (TGF) and fibroblast growth factor 2 (FGF), were expressed, genes encoding molecules such as collagens I and III and pluripotency markers, such as POU5F1 (OCT3/4), SOX2, and NANOG were expressed higher than DPSC [26]. Further, SHED cells exhibited a reduced ability to form neurospheres which was related to the expression of nestin, a marker of neuroepithelial stem cells [49], which is poorly expressed in SHED cells compared with DPSCs [48]. Furthermore, some authors have shown that subpopulations of SHED cells and some stromal elements of the bone marrow were c-kit/CD34+, though these antigens are considered specific markers of hematopoietic lineages [5], which suggest that stem cells can share characteristics despite having different ontogenetic origins [11].

The DPSCs of permanent teeth, impacted third molars, and supernumerary teeth, which today are considered medical waste [50–52], are particularly interesting stem cells. DPSCs recently isolated as the CD90+, CD146+, CD105+, and CD45− cells of supernumerary teeth were found to be capable of multilineage differentiation and were also OCT4+ and NANOG+ [31].

**Figure 1:**
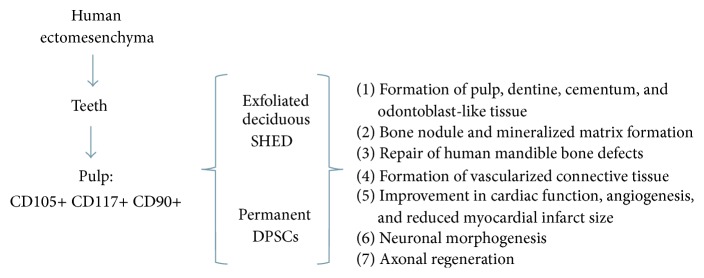
Human DPSC results achieved up to now. DPSCs: stem cell derived from permanent teeth; SHED: stem cell derived from human exfoliated deciduous teeth.

Until recently it was believed that SHED cells, compared with the DPSCs isolated from adult teeth, had advantages for tissue engineering, including their increased proliferation rate [44], which could allow the rapid expansion of these cells in vitro before their reimplantation [53], and the painless nature of collecting these stem cells [23] from tissues regarded as “disposable” and easily accessible [44], which were ideal for young patients who had suffered invasion pulp necrosis in their immature permanent incisors following a trauma [54]. Considering these aspects, Akpinar et al. analyzed the degree of heterogeneity of natal DPSCs, SHEDs, cells and DPSC derived from impacted third molars and found that, regardless of the origin (female baby born with a tooth, a girl who lost a deciduous tooth at the age of six years and one impacted third molar belonging to a 27-year-old man, resp.), these DSCs had similar characteristics in terms of their morphology, rates of proliferation, expression of cell surface markers, and differentiation potentials. Interestingly, flow cytometric analysis indicated that all three types of DSCs were positive for CD13, CD29, CD44, CD73, CD90, CD146, CD166, and HLA-ABC. The results also showed that SHED cells and DPSCs had a similar cell cycle distribution, with most of the cells (78%) in the G1 phase of the cell cycle, which indicate that the cells were growing in size and synthesizing mRNAs and proteins, whereas cells in S- and G2-phases were present in lower proportions (12% and 10%); and the number of cells that were undergoing mitosis was less than that of the actively growing cells [21]. These recently obtained results showed that DPSCs and SHED cells were more similar to each other than originally supposed. Thus, the implementation of these two cell types at the clinical level should provide the same opportunities and the same level of ease of access to autologous stem cells, which is a competitive advantage over that of stem cells derived from other niches, such as bone marrow-derived MSCs [55]. Although more human transplantation assays have been performed using DPSCs compared with those performed using SHED cells, the results obtained were similar (Table 2 and Figure 1).

## 5. DPSC Isolation

Two basic methods for the isolation of DPSCs have been described: (a) the explant method (DPSC-OG) and (b) the enzymatic digestion of pulp tissue method (DPSC-ED). Using the first method, the pulp tissue is surgically removed and the cells are grown from tissue fragments [73–75] whereas, using the second technique, the dental pulp is digested using collagenase and dispase [58, 76], after which the cells are seeded, when cell proliferation is observed, and the MSCs are characterized using flow cytometry based on staining with specific markers [29]. Alternatively, some authors have proposed that the isolation of more immature stem cells requires a tissue explant multistage-process in which the progenitor cells are first grown in culture; subsequently, enzymatic digestion is performed and the isolated cells are expanded [77, 78].

To date, an isolation technique that is superior to the others in terms of proliferative capacity, karyotypic stability, or clinical use of DPSCs has not been found; however, most protocols involve enzymatic digestion rather than systemic explantation and generally the cells to at least one round of in vitro expansion obtain a sufficient number of cells for biomedical use. In this regard, different groups have designed and evaluated increasingly efficient methods for the isolation, expansion, and maintenance of clinically safe human DPSCs in sufficient numbers for various protocols [78]; for example, 1 × 10^6^ cells are needed for xenotransplantation into immunodeficient mice, to retain their capacity for regeneration and differentiation with a minimum of any potential risk. Unfortunately, much about the physiology of DPSCs and how these protocols could be optimized is unknown.

## 6. DPSC Culture

The initial growth period of MSCs on a plastic surface is characterized by the formation of colonies derived from a single cell. The colonies generated in primary culture can be subcultured, typically through multiple passages, because these cells have a strong tendency to expand in culture [79].

Interestingly, studies have shown that DPSCs can be grown for long periods without compromising their plasticity and ability to form bone nodules in vitro. Suchánek et al. showed that DPSCs achieved 60 population doublings in culture medium designed for bone marrow whereas Laino et al. accomplished 80 passages by maintaining the DPSCs-substrate interaction and cell-cell communication in the central region of the secreted extracellular matrix [22, 29]. Interestingly, it has been reported that after reaching the maximum number of passages before entering senescence (the Hayflick limit), DPSCs still had a normal karyotype and doubling period at up to 40 doublings, which was between 12 and 50 hours but which increased to 60 to 90 hours after 50 replications [80]. However, it is generally accepted that a prolonged expansion period may induce senescence and that after 20 to 40 population doublings, MSCs may lose some biological activities (potency). Thus, one of the most important issues regarding DPSCs, and in general for all cells with potential clinical use, is the number of in vitro passages that is most favorable to allowing an in vivo therapeutic effect. The information available for DPSCs is limited, but it has been shown, at least for the treatment of GVHD using MSCs, that the number of cells in fresh tissue (bone marrow or adipose tissue) was not sufficient for therapy. The vast majority of clinical trials exploring the ex vivo expansion of MSCs found that the smallest number of passages, employed for MSC expansion, correlated with a better patient response and a better survival. Thus, von Bahr et al. showed that 75% of patients with GVHD who received MSCs from first and second passage had a one-year survival; however, only 21% of those who received MSCs from third to fourth passages survived [81]. These results agree with those of Choi et al. who compared the therapeutic effect of bone marrow-derived MSCs that had undergone 3, 5, 7, and 9 passages in the treatment of amyotrophic lateral sclerosis (ALS) and found that MSCs of early passages were best suited for therapy because of their stability, their anti-inflammatory properties, and their more powerful neuroprotective effect, compared with those of MSCs with the highest number of passages [80]. Therefore, although the DPSCs of early passages might be more suitable for human therapy, comparative studies of the biological behavior of human DPSCs, obtained from different numbers of passages, are essential to ensure that one type is superior to the others in terms of clinical efficacy.

It is accepted that the proliferation rate of MSCs is highly variable depending on the formulation of the medium (basal media and supplements), the substrate area, the density of cell seeding, and the physical-chemical environment (oxygen and dissolved CO_2_ concentrations, temperature, pH, osmolality, and buffer system). Thus, whereas the cells in a mature tooth pulp are not exposed to a hyperoxic environment, the ambient oxygen tension in conventional cell-cultures ranges from 18% to 21% O_2_ [82, 83]; in contrast, the physiological levels of O_2_ in a tooth range from 3% to 6%. Recently El Alami et al. compared the proliferation rate of human DPSCs cultured under these contrasting atmospheric pressures (21% and 3% O_2_) and found that, under the conventional culture conditions, there was a low rate of proliferation, with oxidative stress and the activation of antioxidants-defense genes such as that of NRF-2, which can interact with proteins related to the cell cycle that play important roles in regulating cell proliferation, thus suggesting that cultivating DPSCs under normal cell-culture oxygen concentrations may not allow obtaining high yields of viable stem cells [84]. Furthermore, protocols for growing human DPSCs involve the use of fetal bovine serum (FBS), the composition of which is unknown and which varies between batches, thus hampering the reproducibility of experiments; however, although the use of FBS is considered to be relatively safe for human therapeutic applications, the use of supplements of nonhuman origin is still a matter of substantial debate [85]. In this sense, there is a risk that nonhuman growth supplements may be contaminated with human pathogens such as viruses, mycoplasmas, prions, or other toxic or immunogenic agents [86–88]. Although these quality-control and biosecurity concerns are more theoretical than real because no such problems have been reported for transplants of DPSCs obtained from cultures that were expanded in serum-containing medium, in response to this risk, various methods of serum-free culture, using chemically defined materials as supplements, have been proposed. Thus, Takeda-Kawaguchi et al. recently used a chemically defined medium (MSCGM-CD) to grow isolated human DPSCs that did not have a reduced colony forming ability as compared with that of cells grown in medium supplemented with FBS and had the ability to differentiate into odontoblasts in vitro and to form dentine-like structures when transplanted into immunodeficient mice [89]. In contrast, some authors have proposed that, pending the availability of completely serum-free defined medium of GMP (“Good Manufacturing Practices”) grade, products derived from secure human blood, such as platelet lysates, might be considered a viable alternative to FBS [90]. Therefore, the conditions for serum-free cell expansion comparable to that obtained using serum-containing medium must be defined in the future, and it is necessary to analyze whether to employ rigorous expansion protocols.

## 7. DPSC Applications

It has been suggested that the dental pulp is a potential source of stem cells for orthopedic, oral, and maxillofacial reconstruction. For example, Yamada et al. demonstrated in canines that MSCs derived from the pulp of the deciduous teeth of puppies and that of adult teeth have the ability to form bone when grafted into the jaw [91]. Although there is a possibility that such cells have applications beyond the scope of the stomatognathic system [33], to date, most of the studies in which human DPSCs, isolated from third molars, or incisors and SHED cells had been transplanted reported the ability of these cells to generate mineralized tissue, extracellular matrix type structures dentine, periodontal ligament, or dental pulp (Table 2).

Interestingly, the general procedure for human DPSC administration is to implement them in scaffold or porous biomaterial to reinforce the graft site and induce tissue regeneration. Various materials have been used to produce scaffolds, including the following: collagen nanofibers (NF) poly-L-lactic acid (PLLA), chitosan, PEGylated fibrin HA/TCP, or a combination of these materials; for example, Ravindran et al. applied DPSCs, PDLSCs, and bone marrow-derived MSCs to collagen/chitosan scaffolds at a density of 2 × 10^6^ cells/scaffold and observed the odontogenic differentiation of DPSC and bone marrow-derived MSCs, without the need to add exogenous growth and differentiation factors [92]. Zhang et al. seeded 2 × 10^6^ DPSCs/per silk fiber/collagen/hexafluoro-2-propanol (HFIP) scaffold and observed that they formed soft dental pulp [93]. Huang et al. created a poly-D, L-lactide, and glycolide (PLG) substance that formed a porous scaffold in which they seed 1 × 10^7^ DPSCs, which resulted in the development of odontoblast cells that produced dentine type material and also had the ability to regenerate pulp type tissue [94].

## 8. DPSCs and Tissue Repair

It is known that MSCs are involved in growth, wound healing, and cell replacement under both physiological and pathological conditions. These cells have been shown to be effective in regenerating periodontal tissue, diabetic critical limb ischemic tissue, bone damage caused by osteonecrosis, skin lesions caused by burns [47, 95, 96], liver, neuronal and skeletal muscle tissue, and blood vessels [97–100] among other tissues [3]. Regarding DPSCs, their ability to differentiate into odontoblasts was a major boon for their use in tooth-tissue engineering, but growing evidence that these stem cells were also capable of repairing extraoral tissues, for example, tissues of the musculoskeletal system, because of their similarities to bone marrow-derived MSCs [51, 91], has resulted in their currently being recognized as one of the most accessible and attractive multipotent MSCs, with high rates of growth, for use in engineering tissue and in regenerative medicine [101].

## 9. Periodontal Regeneration with DPSC

One of the most common chronic infectious disorders, affecting up to 90% of the population worldwide, is periodontal disease (PD) [102]. At the periodontal level, repair involves healing a wound even without completely restoring the original architecture or tissue function. In contrast, regeneration involves reproducing lost or damaged part, so that the original functionality is completely restored. The PD type, called periodontitis, is manifested by the progressive destruction of the structures supporting the teeth and is a major cause of tooth loss in adults [103]. Thus, in the case of periodontitis, periodontal regeneration would involve the complete restoration of all the components of the periodontal ligament, including periodontal and gingival connective tissue, cementum, and alveolar bone, which is a challenge in clinical practice [104], because when histological tests showed good regeneration, the new tissues were similar to the lost tissues but had different characteristics, although, in most cases, regeneration of the periodontal ligament failed between the neoformed tissue and bone cementum [4].

Current regeneration protocols, such as those using autologous bone grafts, allografts, or alloplastic materials, also have limitations because they generally result in tissue repair but not in true regeneration [105] and cannot be used in all clinical situations [106]. Thus, these protocols, although promising, are far from a medical certainty. Other procedures such as the addition of growth and differentiation factors [107] and anti-inflammatory molecules [108, 109] yielded positive results by inducting periodontal regeneration, but the average half-life of these factors is short, which limits their use in regenerative therapy.

Preclinical studies have shown that DPSCs, isolated from human third molars and transplanted into immunodeficient mice and rats, differentiated into cementoblast-like cells, adipocytes and collagen forming cells with the ability to generate material similar to periodontal tissue cement [105]. The expression of STRO-1, CD146, and CD44 has been observed in cells involved in periodontal regeneration, and Du et al. showed that SDF-1 was an additional indicator of periodontal tissue regeneration [109, 110].

Studies have shown that it is possible to form complex structures such as pulp-dentine, root cementum, and the periodontal ligament, by transplanting DPSCs into immunocompromised mice (Table 2), and that these cells may be involved in the repair processes that occur within periodontal defects created in rodents. There are also reports showing that SHED cells are able to stimulate bone formation, which raises the possibility that they could be used to induce bone craniofacial bone regeneration [111]. However, although these and other experimental animal data provided clear evidence of the potential of DPSCs to induce the formation of dental tissues, clinical trials using DPSCs have not been widely reported [39]. D'Aquino et al. show that human autologous DPSC/collagen sponge biocomplex implants restore the mandibular bone tissue of patients [51].

Interesting is that clinical assessment, conducted 3 years after implantation of autologous human DPSC, has shown that functionality of oral cavities, mandibles, and mucosal membranes was normal and no alterations were observed; however, a thorough histological study and an advanced in-line holotomography revealed that regenerated tissue from the graft sites was composed of a fully compact bone uniformly vascularized and with a high matrix density, rather than a spongy type that is physiological for the area; thus, the bone regenerated was completely different from normal alveolar bone. Authors suggest that this is probably because grafted DPSCs do not follow the local signals of the surrounding spongy bone [72]. Recently it was found that pretreatment of DPSCs with valproic acid (VPA), a selective inhibitor of histone deacetylases (HDAC), significantly improves mineralized matrix formation, increasing the expression of bone glycoproteins involved in the formation of the mineralized matrix and negatively affecting late-stage markers of differentiation [112]. This suggests that regulating the response of stem cells to epigenetic changes may be the key to regeneration with DPSCs, successfully generating complex structures (a true regeneration), and not only does it bring a tissue that resembles the original, such as what is achieved using the tissue engineering strategies available today. Whether, as suggested by Jo et al., application of epigenetic regulators, for example, HDAC inhibitors [34], may be valuable for stem cell-based interventions [112] is an issue that deserves much more attention in the future.

## 10. DPSCs and Nervous System

Neural crest-related stem cells can be isolated from adult mammalian craniofacial tissues, such as dental pulp [113], periodontal ligaments [114], and salivary glands [115] and D'Aquino et al. provide evidence that adult dental follicle comprises cells having characteristics of neural crest cells or, at least, displaying the same pluripotency as those cells, because they easily and efficiently gave rise to neurons, osteoblasts, adipocytes, and other cytotypes and coexpress early neural progenitor markers and vascular endothelial growth factor receptors, such as Brn3a and flk-1, and retain embryonic markers Oct-4, Nanog, TRA1-60, and TRA-1-80-1 [51]. This evidence that cells derived from dental tissue have the skill to give rise to cells of the nervous system can explain that DPSCs have regenerative potential in the damaged central nervous system and replace lost neurons through differentiation, at least in mice [69, 116], as well as the ability to produce neurotrophic factors (NTFs), which promote neuronal survival and axonal guidance; these properties have been attributed in part to their ontogenic neural-crest origin [117, 118].

DPSC significantly improved the survival rate of cultured neurons that were positive for embryonic tyrosine hydroxylase via the release of neurotrophic factors. In animal studies, grafting dental pulp into hemisected spinal cords increased the number of surviving motor neurons [117], and DPSCs were shown to express nestin and glial fibrillary acidic protein (GFAP), at both the gene and the protein level [9].

DPSCs have been shown to promote cell survival and neuritogenesis in in vitro coculture with primary adult rat retinal cells and to promote the survival of retinal ganglion cells (RGCs) and axonal regeneration in an in vivo model of optic nerve crush (ONC). These proregenerative and neuroprotective effects have been attributed primarily to the ability of DPSCs to induce the expression of neurotrophins, including the following: nerve growth factor (NGF), brain-derived neurotrophic factor (BDNF), and neurotrophin-3 (NT-3). However, it has been suggested that retinal glial cells may also contribute to the DPSC-induced neuroprotection [118].

The loss of light sensitive photoreceptors is a major cause of untreatable blindness, and MSC transplantation is a potential strategy to restore vision through restoring photoreceptor function. Thus, various studies have shown that stem cells derived from different sources and progenitor cells can differentiate into retinal photoreceptors both in vitro and in vivo. In this regard, Bray et al. recently showed that DPSCs were responsive to signals from the retina and expressed markers of mature photoreceptors, such as BDNF and rhodopsin, a transmembrane protein of the retinal rods, which reinforces the hypothesis that these cells could be driven to differentiate into functional photoreceptors and raises the possibility of a DPSC-based therapy to repair retinal damage [25].

## 11. DPSCs and Therapy of Immune Disorders

DSC has immunomodulatory ability comparable to that of BM-derived MCS, which is why they have been proposed to be an alternative to MSCs for therapy. Indeed, there is strong evidence showing that dental MSCs may be therapeutic agents for inducing immunosuppression in cases of oral inflammation and even in other chronic inflammatory conditions [3].

## 12. Health Regulations

The use of the DPSCs in extensive clinical applications is not regulated in all countries. In this sense, with the exception of hematopoietic stem cells, authors such as Benitez have noted that Mexico is considered a destination for the application of cell therapies to miraculous treatments, due to the lack of legislation and regulations that would provide security to this new branch of medicine [119].

## 13. Conclusion

In the last decade, significant advances have been made in MSC research; however, currently, there are pending issues that are crucial to the realization of their clinical applications, such as establishing quality-control protocols and security checks. In addition, more research is needed to clarify the source and number of passages of DPSCs that are best suited to induce periodontal regeneration, which route of administration and the type of scaffold is the most appropriate whether there are contraindications for their clinical use and once administered, which parameters should be considered to monitor their clinical efficacy. Therefore, DPSC therapy is promising but as stated by Wei et al., as is the case for MSC therapy in general, it is far from being a mature clinical technology [120].

## Figures and Tables

**Table 1:** Variants of human DPSCs immunophenotype.

Reference	Immunophenotype
Akpinar et al. [21]	CD13+, CD29+, CD44+, CD73+, CD90+, CD146+, CD166+
Suchánek et al. [22]	CD29+, CD44+, CD63+, CD73+, CD90+, CD166+, CD45−, CD34−
Kawashima [23]	CD29+, CD44+, CD73+, CD105+, CD166+, CD14−, CD34−, CD45− *Stro-1+, CD29+, CD44+, CD73+, CD90+, CD105+, CD146+, CD166+, CD271+, CD34+, CD117+, OCT-3/4+, NANOG+* ^*∗*^
Werle et al. [24]	CD29+, CD73+, CD90+, CD14−, CD34−, CD45−, HLA-DR−
Bray et al. [25]	CD44+, CD73+, CD90+, CD105+, CD14−, CD31−, CD45−, HLA-DR−
Govindasamy et al. [26]	CD44+, CD73+, CD90+, CD166+, CD34−, CD45−, HLA-DR−
Lindemann et al. [27]	CD73+, CD90+, CD29+, CD14−, CD34−, CD45−, HLA-DR−
Pivoriūnas et al. [28]	CD73+, CD90+, CD105+, CD146+, CD14−, CD34−, CD45−
Laino et al. [29]	CD90+, CD34+, CD45−
Lindroos et al. [30]	CD90+, CD105+, CD146+, CD9+, CD10+, CD44+, CD49+, CD106+, STRO-1+
Shoi et al. [31]	CD90+, CD105+, CD146+, CD45−
Ishkitiev et al. [32]	CD117+, Oct3/4+, NANOG+
Yang et al. [33]	CD117+, CD34+, CD45−
Jo et al. [34]	STRO-1+, CD29+, CD44+
Liu et al. [35]	STRO-1+, CD29+, CD34+, CD44+, CD106+, CD146+
Dissanayaka et al. [36]	STRO-1+, CD146+, NANOG+, CD73−, CD105−, CD45−
Kerkis et al. [37]	OCT-4+, NANOG+, SSEA-3+, SSEA-4+, TRA-1-60+, TRA-1-81+

^*∗*^DPSCs did not express all of these surface markers or they may have been expressed in different proportions [23].

**Table 2:** Transplantation assays using MSCs obtained from human tooth sources (DPSCs & SHED cells).

Source/scaffold	Host	Cells	Weeks	Results	Reference
DPSC/HA-TCP	I.Mice	5 × 10^6^	6	Formation of pulp, dentine, and odontoblast-like tissue	Gronthos et al. [53]
DPSC/HA/TCP	I.Mice & swine	4 × 10^6^	12	Formation of pulp tissue, dentine, and cementum	Sonoyama et al. [20]
DPSC/NF-PLA	I.Mice	1 × 10^6^	8	Generation of odontoblast-like cells and a collagen-like matrix	Wang et al. [56]
DPSC/fibrin	Mice	1 × 10^6^	4	Formation of dentine-like tissue	Chun et al. [57]
DPSC/NF	I.Mice	1 × 10^5^	4	Mineralized tissue formation	Chen et al. [2]
DPSC/chitosan/collagen	I.Mice	1 × 10^6^	4	Mineralized tissue formation	Yang et al. [33]
DPSC/HA/TCP	I.Mice	5 × 10^7^	12	Formation of dentine-like matrix	Sun et al. [58]
DPSC-3M/HA/TCP	I.Mice	4 × 10^6^	2, 4, 8, and 16	Differentiation into osteoblasts & odontoblast-like cells on the surface of HA/TCP scaffold	Batouli et al. [59]
DPSC-3M/ TCP	SCID mice	1 × 10^6^	8–15	Generation of dentine pulp-like structure	Takeda et al. [60]
DPSC-3M/PLGA	I.Rats	1 × 10^6^	4–8	Bone nodule formation	Graziano et al. [61]
DPSC-3M	Nude rats	1 × 10^6^	2–4	Improvement in cardiac function, angiogenesis, and reduced myocardial infarct size	Gandia et al. [62]
DPSC-3M/HA	I.Mice	1 × 10^6^	8	Mineralized tissue formation	Ikeda et al. [63]
DPSC-3M/PLGA	SCID mice	1 × 10^7^	12–16	Formation of pulp-like tissue	Huang et al. [52]
DPSC-3M/dentine	I.Mice	1 × 10^6^	4	Mineralized tissue formation	Demarco et al. [64]
DPSC-3M/Ceramic/bone	I.Mice	5 × 10^6^	6	Formation of dentine, pulp-like tissue	Wang et al. [56]
DPSC-3M/HFIP/silk	I.Mice	4.5 × 10^5^	6, 18, and 25	Formation of soft pulp-like tissue	Yang et al. [33]
DPSC-3M/HA/TCP	I.Mice	1 × 10^7^	6 & 12	Formation of pulp-like structure lined with odontoblast-like cells	Lee et al. [65]
DPSC-3M/fibroin	Sprague-Dawley rats	1500/mm^3^	4	Bone formation	Riccio et al. [66]
DPSC-3M	I.Mice	—	8	Generation of pulp-like & periodontal ligament type tissue	Lei et al. [67]
DPSC-3M/dentine	I.Mice	1 × 10^4^	4, 6, and 8	Mineralized tissue and dentine formation	Tran and Doan [68]
DPSC-3M & SHED	Avian embryos	5 × 10^3^	24, 48, and 72 h	Neuronal marker expression and neuronal morphogenesis	Arthur et al. [69]
DPSC-3M & SHED	Sprague-Dawley rats	1 × 10^6^	8	Axonal regeneration and inhibition of apoptosis in neurons, astrocytes, and oligodendrocytes	Sakai et al. [46]
SHED/collagen	Wistar rats	1 × 10^6^	1, 3, 4, and 8	Dense and mature bone formation	de Mendonça Costa et al. [70]
SHED/PLA	SCID mice	5 × 10^6^	3	Solid training in dental tissue samples	Sakai et al. [46]
SHED/FPOL	I.Mice	5 × 10^5^	5	Formation of vascularized connective tissue	Galler et al. [71]

DPSC-3M/collagen	Human	—	52	Formation of bone in the extracted socket and repair of periodontal tissue	D'Aquino et al. [51]
DPSC-3M/collagen	Human	—	186	Repair of human mandible bone defects with compact and uniformly vascularized bone	Giuliani et al. [72]

DPSCs: derived from permanent teeth; 3M: third molar; SHED: derived from human exfoliated deciduous teeth; I.Mice: immunodeficient mice; SCID: severe combined immunodeficiency; FPOL: PEGylated fibrin; NF: nanofibrous: PLA: poly(L-lactic-acid); HFIP: hexafluoro-2-propanol; PLG: poly-D,L-lactide and glycolide; HA/TCP: hydroxyapatite/tricalcium phosphate; AT: autologous transplant.
